# Low-Intensity Running and High-Intensity Swimming Exercises Differentially Improve Energy Metabolism in Mice With Mild Spinal Muscular Atrophy

**DOI:** 10.3389/fphys.2019.01258

**Published:** 2019-10-01

**Authors:** Léo Houdebine, Domenico D’Amico, Jean Bastin, Farah Chali, Céline Desseille, Valentin Rumeau, Judy Soukkari, Carole Oudot, Thaïs Rouquet, Bruno Bariohay, Julien Roux, Delphine Sapaly, Laure Weill, Philippe Lopes, Fatima Djouadi, Cynthia Bezier, Frédéric Charbonnier, Olivier Biondi

**Affiliations:** ^1^UMR-S1124, INSERM, Faculté des Sciences Fondamentales et Biomédicales, Université Paris Descartes, Paris, France; ^2^Biomeostasis CRO, Nutritional Behavior and Metabolic Disorders, La Penne-sur-Huveaune, France; ^3^UFR STAPS, Université d’Evry Val-d’Essonne, Evry, France; ^4^Biophytis, Sorbonne Université, Paris, France

**Keywords:** Spinal Muscular Atrophy, energy metabolism, physical exercise, fat oxidation, oxygen consumption, muscle mitochondria, respiratory chain

## Abstract

Spinal Muscular Atrophy (SMA), an autosomal recessive neurodegenerative disease characterized by the loss of spinal-cord motor-neurons, is caused by mutations on Survival-of-Motor Neuron (SMN)-1 gene. The expression of *SMN*2, a *SMN1* gene copy, partially compensates for *SMN1* disruption due to exon-7 excision in 90% of transcripts subsequently explaining the strong clinical heterogeneity. Several alterations in energy metabolism, like glucose intolerance and hyperlipidemia, have been reported in SMA at both systemic and cellular level, prompting questions about the potential role of energy homeostasis and/or production involvement in disease progression. In this context, we have recently reported the tolerance of mild SMA-like mice (*Smn^Δ7/Δ7^; huSMN2*^+/+^) to 10 months of low-intensity running or high-intensity swimming exercise programs, respectively involving aerobic and a mix aerobic/anaerobic muscular metabolic pathways. Here, we investigated whether those exercise-induced benefits were associated with an improvement in metabolic status in mild SMA-like mice. We showed that untrained SMA-like mice exhibited a dysregulation of lipid metabolism with an enhancement of lipogenesis and adipocyte deposits when compared to control mice. Moreover, they displayed a high oxygen consumption and energy expenditure through β-oxidation increase yet for the same levels of spontaneous activity. Interestingly, both exercises significantly improved lipid metabolism and glucose homeostasis in SMA-like mice, and enhanced oxygen consumption efficiency with the maintenance of a high oxygen consumption for higher levels of spontaneous activity. Surprisingly, more significant effects were obtained with the high-intensity swimming protocol with the maintenance of high lipid oxidation. Finally, when combining electron microscopy, respiratory chain complexes expression and enzymatic activity measurements in muscle mitochondria, we found that (1) a muscle-specific decreased in enzymatic activity of respiratory chain I, II, and IV complexes for equal amount of mitochondria and complexes expression and (2) a significant decline in mitochondrial maximal oxygen consumption, were reduced by both exercise programs. Most of the beneficial effects were obtained with the high-intensity swimming protocol. Taking together, our data support the hypothesis that active physical exercise, including high-intensity protocols, induces metabolic adaptations at both systemic and cellular levels, providing further evidence for its use in association with SMN-overexpressing therapies, in the long-term care of SMA patients.

## Introduction

Spinal Muscular Atrophy (SMA) is an autosomal recessive neurodegenerative disease characterized by the specific loss of spinal cord motor neurons (MNs) which induce progressive muscular atrophy and could lead to patient death when respiratory muscles are affected (Crawford and Pardo, 1996). SMA is mainly caused by mutations in the telomeric copy of the Survival of Motor Neuron (*SMN*) genes, named *SMN1* (Lefebvre et al., 1995). *SMN2*, the SMN centromeric gene copy, only partially compensates the lack of *SMN1* expression because the exon 7 is excluded in 90% of transcripts, leading to the production of an unstable SMN protein. Consequently, the number of *SMN2* genes and their expression levels are directly linked to SMA clinical severity, classified from the most severe type 1 to the mild form type 3, based on the age of onset and on disease progression (Harding and Thomas, 1980).

Although the molecular origin of neurodegeneration in SMA is established in the vast majority of cases, i.e., a depletion of SMN protein in MNs leading to their degeneration, the physiopathology of the disease is today considered to be much more complex than initially thought. Noteworthy, SMN protein has a largely ubiquitous expression and is involved in mRNA metabolism. Thus, SMN-depletion induced defects have been reported in many different tissues in addition to the central nervous system and, independently of MN death, notably in the heart (Finsterer and Stollberger, 1999; Bevan et al., 2010; Heier et al., 2010; Shababi et al., 2010; Biondi et al., 2012), vasculature (Somers et al., 2016), skeletal muscles (Braun et al., 1995; Cifuentes-Diaz et al., 2001; Nicole et al., 2003; Biondi et al., 2008), pancreas (Bowerman et al., 2012, 2014) and liver (Vitte et al., 2004; Sahashi et al., 2013). Interestingly, pancreas and liver are directly involved in energy metabolism regulation, vasculature in tissue-oxygenation and heart and skeletal muscles are the main energy consumers in the body. These observations prompted to study in patients and mouse models energy metabolism state in SMA and their potential role in the pathophysiology. Altogether, these data pointed out profound alterations in the main catabolic pathways, including glycolysis (Bowerman et al., 2012; Davis et al., 2015) and fatty acid oxidation (Tein et al., 1995; Crawford et al., 1999). Furthermore, these defects could also be associated with severe perturbations in insulinemia (Davis et al., 2015) and glucose tolerance (Bowerman et al., 2012; Davis et al., 2015). At the cellular level, fatty acids and carbohydrates fuel mitochondria, the most important provider of energy in eukaryotic cells, through the functioning of the respiratory chain in the mitochondrial inner membrane that leads to efficient ATP production. In energy voracious tissues such as skeletal muscles, the maintenance of the mitochondrial network, qualitatively and/or quantitatively, is crucial to adapt to the workload requested for setting up moving or breathing. Interestingly, mitochondrial dysfunctions have been reported in SMA muscles, with alterations in the muscular mitochondrial biogenesis (Ripolone et al., 2015) and in the expression levels of respiratory chain components (Sperl et al., 1997; Jongpiputvanich et al., 2005; Miller et al., 2016).

Following the introduction of adequate clinical care and SMN-restoration therapies in MNs, such as Nusinersen, SMA patients are living longer (Chiriboga et al., 2016; Hache et al., 2016; Finkel et al., 2017). However, SMN expression is still not enhanced in all the affected tissues. Therefore, it appears of paramount importance to find efficient ways to induce whole-body adaptations in order to limit the potential impact of metabolic impairments, to improve muscle resistance to fatigue and to personalize the clinical care for the long-term quality of life of patients. In this context, physical exercise is expected to efficiently improve muscular energy metabolism and consequently limit muscle fatigue, with subsequent whole-body glycemic benefits, even in case of insulin sensitivity impairments, glucose resistance (Wojtaszewski et al., 2000; Cunha et al., 2015; Naufahu et al., 2018), and perturbations in lipids metabolism (Pistor et al., 2015; Wang et al., 2017; Mika et al., 2019). However, despite several recent trials (Lewelt et al., 2015; Madsen et al., 2015; Montes et al., 2015; Bora et al., 2018; Bartels et al., 2019), the use of physical exercise in SMA patient care is still under debate and no data concerning the potential impact of exercise on SMA-induced metabolic defects are available to date. Thus, additional studies directly addressing the potential benefits provided by different types of physical exercise on the energetic metabolic state in SMA are highly warranted.

In the present work, we analyzed the metabolic adaptations of mild SMA-like mouse (*Smn^Δ7/Δ7^; huSMN2*^+/+^) population exposed to two different types of long-term exercises, i.e., a low-intensity running- or a high-intensity swimming-based training, which we previously showed efficient to improve several SMA hallmarks in an SMN-expression independent manner, including MN death, muscle atrophy and locomotor behavior (Chali et al., 2016). Here, (Deforges et al., 2009) we report that several SMA-induced systemic metabolic defects, notably glucose homeostasis impairments and lipid overload, were significantly improved by physical exercise. These benefits were associated in particular with an improvement in fast-twitch SMA muscle mitochondrial efficiency. Furthermore, each exercise paradigm provided differential effects on SMA muscle metabolism calling for personalized exercise designs when transferring these data to patients more so when knowing the diversity of SMA patient metabolic state.

## Materials and Methods

### Mild SMA-Like Mouse Model

The knockout-transgenic mild SMA-like mice (FVB/NRj-Smn^Δ7/Δ7^, *huSMN2*^+/+^) derived from mice obtained from the Institute of Molecular Biology (Hsieh-Li et al., 2000) (Academia Sinica, Taipei, Taiwan) have been purified on the FVB/NRj genetic background (Janvier Labs, Le Genest-Saint-Isle, France) by backcross for more than 10 generations and were designated as ‘SMA’ (*n* = 53). The control mice (CTRL; *n* = 53) were heterozygous knock-out for murine *Smn*, expressing homozygous human *SMN2* transgene (FVB/NRj-*Smn*^+/Δ7^, *huSMN2*^+/+^). Only males were selected for experimentation to standardize the analyses, and all the experiments were performed in a blind systematic manner to minimize bias. From two to four animals were housed in each cage, with food and water *ad libitum*. Animal handling and experimentation were performed in line with approved Institutional Animal Care and Use Committee protocols at the University of Paris Descartes (CEEA 34, agreement number B75-06-07) and followed the national authority (Ministere de la Recherche et de la Technologie, France) guidelines for the detention, use and the ethical treatment of laboratory animals based on European Union Directive 2010/63/EU.

### Exercise Protocols

Two-month-old mice were submitted to 10 consecutive months of training either to running-based exercise on a speed-regulated treadmill at 13 m.min^–1^, or to swimming-based exercise in an adjustable-flow swimming pool at 5 L.min^–1^ as previously described (Chali et al., 2016). Sedentary control and SMA (35 Sed CTRL and 35 Sed SMA) mice were placed in the pool without flow (18 SMA and 18 CTRL mice) and floated at the water surface or on the treadmill without speed (17 SMA and 17 CTRL mice). We formed four groups of trained mice, one running group of controls and one of SMA (Run CTRL and Run SMA; *n* = 18 for each) and one swimming group of controls and one of SMA (Swim CTRL and Swim SMA; *n* = 18 for each).

### Glucose Homeostasis Evaluation

An Oral Glucose Tolerance Test (OGTT) and an Insulin Tolerance Test (ITT) were performed on sedentary groups of mice at 3, 6, and 12 months of age and on trained groups of mice at 6 and 12 months. The two tests were performed on each mice with a delay of 1 week between each. Briefly, overnight fasted mice received an oral load of glucose (2 g.kg^–1^) or received an intraperitoneal injection of insulin (0.75IU.kg^–1^; Actrapid,^TM^ Novonordisk). A drop of blood was collected from tail tip just prior the administration (T0) and 5, 20, 60, 90, and 120 min following glucose ingestion or insulin injection and glycemia was determined via a glucometer (Accu-Chek^®^ performa glucometer, France).

### Measurement of Body Weight, Food Intake, Respiratory Exchanges and Related Parameters

Body weight was measured daily, before the onset of the light phase. At 12 months of age, and 48 h after the last training session, food intake, total spontaneous activity, oxygen consumption (VO_2_) and carbon dioxide production (VCO_2_) were measured for each mouse, individually, during 2 consecutive days (D1 and D2) by using the Oxylet Physiocage System (Panlab/Harvard apparatus, Cornella, Spain), the software suite METABOLISM (V2.2.01, Panlab) and the obtained data were represented as a mean of the 2 days. Mice were habituated to the metabolic chambers for 24 h before data collection.

The respiratory exchange ratio (RER) was calculated as VCO_2_/VO_2_, Energy Expenditure (EE) was calculated according to the formula: EE (kcal.day^–1^.kg^–0.75^) = VO_2_ × 1.44 × [3.815 + (1.232 × RER)], the fat oxidation (Fat ox.) was calculated according to the formula: Fat ox. (g.day^–1^.kg^–0.75^) = (1.72 × VO2) – (1.72 × VCO2) – (1.96 × 0.2) and the carbohydrate oxidation (CH ox.) was calculated according to the formula: CH ox. (g.day^–1^.kg^–0.75^) = (−2.97 × VO_2_) + (4.17 × VCO_2_) – (2.44 × 0.2).

The analyses of total spontaneous activity, respiratory exchanges and related parameters were also analyzed by dividing the day in 4 periods of 6 h: two periods (0–6 h and 6–12 h) during the diurnal phase and two periods (12–18 h and 18–24 h) during the nocturnal phase.

Finally, the mice EE was evaluated during minimal (Min) and maximal (Max) activity in diurnal and nocturnal phases. Briefly, the analyses of the total spontaneous activity, expressed in arbitrary unit (a.u), were performed by plotting data every 7 min 30. We defined a minimal activity as an activity below 10 a.u over a period of 7 min 30 and a maximal activity as an activity up to 100 a.u. over a period of 7 min 30. A Min EE was represented as a mean of each EE related to a minimal activity during diurnal or nocturnal phases. In contrast, a Max EE was represented as a mean of each EE related to a maximal activity during diurnal or nocturnal phases.

### Insulin ELISA

Blood samples were collected from right ventricle on anesthetized mice, with 1% pentobarbital solution (6 μL.g^–1^) diluted in 0.9% saline buffer, using a 1 mL syringe mounted with a 22-gauge needle and coated with heparin (5000 UI/mL, Panpharma Luitré, France) in resting condition for all group of mice (24 h after training). Blood samples were centrifuged (1000 *g*, 10 min, + 4°C) and serum was frozen at −80°C. The serum insulin level was measured using a rat/mouse ELISA assay kit (EZRMI 13K, Millipore) following manufacturer’s instructions. For each mouse, 10 μL of serum were incubated with biotinylated anti-insulin antibody at room temperature for 2 h in agitation at moderate speed, about 400–500 rpm followed by 30 min incubation with streptavidin-horseradish peroxidase conjugate buffer. Insulin concentration was determined reading on spectrophotometer at 590 nm wavelength and 450 nm wavelength. Insulin levels were given in nmol/mL.

### Thermoregulation Assessment

The thermoregulation of mice was assessed under two thermal stress tests: the cold air test (+12°C) and the cold water test (+10°C). For the cold air test, mice were individually housed in an air-conditioned room at +12°C and rectal temperature was recorded every 20 min until 2 h using a rodent rectal temperature probe (BIO-9882 Dual Input Thermometer, Bioseb, Vitrolles, France). For the cold water test, mice were individually put in plastic cage with 4 cm of 10°C water in order to recover the entire body of mice avoiding swimming. Rectal temperature was recorded every 10 min for 1 h.

### Muscles Transmission Electron Microscopy

*Tibialis* and *soleus* muscles of 12-month-old mice were dissected immediately after cervical dislocation, divided in pieces with blade then were fixed and stored with a 4% paraformaldehyde solution supplemented with 2.5% glutaraldehyde in 0.1M phosphate buffer at pH 7.4. Pieces of muscles were then supported by the electron microscopy platform of Cochin institute for all processes and imaging, as follows. Pieces of muscle were washed in phosphate buffer, postfixed in 1% osmium tetroxide, dehydrated in graded ethanol series, and embedded in epoxy resin. Ultrathin muscles sections (80–90 nm) were cut longitudinally on an ultramicrotome (UC-7, Leica) and collected on 200-mesh nickel grids. Staining was performed on drops of 4% aqueous uranyl acetate, followed by Reynolds’s lead citrate (Reynolds, 1963). Mitochondrial ultrastructural analyses were performed in a JEOL jem-1011 electron microscope and digitalized with DigitalMicrograph software on submembranar and intramyofibrillar mitochondria.

### Muscles Proteins and Western Blot Analysis

Twelve-month-old mice were anesthetized with intraperitoneal injection of pentobarbital 1% (6 μL.g^–1^). *Tibilias*, *plantaris* and *soleus* muscles dissected, immediately frozen in liquid nitrogen and homogenized in 100 μL per 5 mg tissues in the presence of ice-cold RIPA buffer [50 mM Tris, 150 mM NaCl, 0.1% SDS, 1% NP40, 10 mM NaF, 1X protease inhibitor (Roche, Basel, Switzerland), 1% phosphatase inhibitor (Sigma-Aldrich, St. Louis, MO, United States)] using metal beads in 2 mL tubes and mechanically stressed with a TissueLyser II apparatus (Qiagen ID85300, United States). Protein concentration of the clarified homogenates (+4°C, 20 min, 17,000 g) was determined on all samples using the Lowry protein assay (Lowry et al., 1951). Whole cell extracts [15 μg VDAC1/Porin, Cyclophilin D or 10 μg (Oxphos)] were fractionated by 12.5% SDS-PAGE(1.5 M Tris pH 8.3, 12.5% acrylamide, 0.07% Bis, 0.1% SDS, 0.05% ammonium persulfate, 0.06% tetramethylethylenediamine). The separated proteins were transferred on PVDF membranes (Bio-Rad Laboratories) according to the Towbin protocol (Towbin et al., 1984). Equal loading of samples was checked by Ponceau dye staining of the transferred gels. Western blot analysis was performed on membranes overnight at +4°C in 4% BSA, 0.05% Tween 20, TBS pH 7.4. Each of the following primary antibodies, including, mouse anti-total OXPHOS rodent cocktail (1:1000; Abcam, ab110413), mouse anti-VDAC1/Porin (1:2000, 36 kDa, Abcam, ab14734), mouse anti-alpha Tubulin (1:10000, 50 kDa, Abcam, ab7291), anti-cyclophilin D (1:1000, 18 kDa, MitoSciences, MSA04), was incubated overnight at +4°C in the above blocking medium. Membranes were rinsed in 0.1% Tween 20 in TBS three times for 10 min each time at room temperature and then incubated in horseradish peroxidase-conjugated goat secondary antibody directed against mouse Igs (1:5000; Bio-Rad Laboratories) and in horseradish peroxidase-conjugated goat secondary antibody directed against rabbit Igs (1:10,000; Jackson ImmunoResearch) in 0.1% Tween 20 in TBS for 1 h at room temperature. Bound antibody complexes were developed with AmershamTM ECL^TM^ Western Blotting Analysis System (GE Healthcare, Bio-Science, Upsala, Sweden). In some instances, membranes were stripped after immunoblotting by incubation in stripping buffer (100 mM-mercaptoethanol, 2% SDS, and 62.5 mM Tris-HCl, pH 6.7) for 30 min at +55°C with agitation, and membranes were then blocked and reprobed with monoclonal mouse anti-glyceraldehyde-3-phosphate dehydrogenase antibody (GAPDH) (1:1000, 37 kDa, Millipore, MAB374). Images were done using ImageQuant LS4000 (GE Healthcare Bio-Science, Upsala, Sweden) and quantification performed using Image J v1.52i software.

### Isolated Mitochondrial Oxygen Consumption

*Tibialis* and *plantaris* muscles were dissected after cervical dislocation of 12-month-old mice and transferred in a glass cupule containing ice-cold extraction buffer pH 7.2 (20 mM Tris-HCl, 250 mM sucrose, 2 mM EDTA, and 40 mM KCl). The muscles were immediately weighed, finely minced all together with scissors in 1 mL of extraction buffer supplemented with 1 mg.mL^–1^ BSA (medium A), and homogenized immediately by 10–15 strokes at 500 rpm with a motor-driven Teflon/glass homogenizer. The homogenate was filtered through a 90 μm nylon gauze and centrifuged at 1000 *g* for 5 min at +4°C. The supernatant was centrifuged at 14000 g, +4°C, for 10 min, and the pellet containing mitochondria was resuspended in 1 mL of medium A supplemented with 5% Percoll, and centrifuged at 14000 *g* for 10 min. The pellet of washed mitochondria was carefully resuspended in medium A with 5% Percoll on ice, by adjusting the resuspension volume with a ratio of 1.33 μL.mg^–1^ of muscle wet weight. Measurements of mitochondrial oxygen consumption rates were performed right after isolation using the Oxoplate technology, based on 96-well microplates with integrated optical oxygen sensors (PreSens, Germany). Each well was filled with 100 μL of incubation medium containing 20 mM KH_2_PO_4_/K_2_HPO_4_ pH 7.4, 300 mM Mannitol, 10 mM KCl, 5 mM MgCl_2_, and either 1 mM pyruvate + 2 mM malate or 37 μM palmitoy-CoA + 2 mM malate + 1 mM carnitine, pre-warmed at +37°C. Then, 5 μL of purified mitochondria were added in each well, which were covered with 200 μL of mineral oil to isolate from ambient oxygen. Oxoplates^®^ were then read out from the bottom every 30 s for 20 min by a fluorescence intensity microplate reader (infinite^®^M200, Tecan). The kinetics of fluorescence intensities were analyzed according to the manufacturer’s instruction manual, and the oxygen consumption assessed by determining the maximal slope of decrease of oxygen partial pressure relative to 100%, at +37°C. The results were finally expressed as percent of O_2_ per minute per mg of mitochondrial proteins. Total mitochondrial protein content was determined by the Lowry method (Lowry et al., 1951).

### Respiratory Chain Activity

Twelve-month-old mice were anesthetized by intraperitoneal injection of pentobarbital 1% (6 μL.g^–1^ body weight). *Tibialis*, *plantaris* and *soleus* muscles were dissected, immediately frozen in liquid nitrogen and stored at −80°C. Frozen muscles were weighed and homogenized in cold extraction buffer (1/25 weight/volume) using motor-driven Teflon/glass homogenizer, and the homogenates were centrifuged at 1000 *g* for 10 min at +4°C. The supernatants were then frozen at −80°C and used within 2 months for enzyme determinations. Total protein content of homogenates was determined by the Lowry method (Lowry et al., 1951).

The methods used for measurements of respiratory chain (RC) complex I and II activities, based on following the decrease in absorbance of dichloroindophenol (DCIP), were adapted from the method developed by Janssen et al. (2007). RC complex I activity was measured at the spectrophotometer at 614 nanometer (nm) in 1 mL of 25 mM potassium phosphate buffer pH 7.8 at 37°C, containing 3.5 mg/ml BSA, 70 μM DCIP, 90 μM decylubiquinone, and 0,2 mM NADH. The reaction was started by addition of 10–15 μL of muscle homogenate and the slopes were recorded continuously for 1–2 min before and after addition of 10 μM rotenone. CI activity was calculated as the rotenone sensitive fraction, and expressed as nanomol (nmol) DCIP reduced.min^–1^.mg^–1^ protein. RC complex II activity was immediately measured in the same cuvettes by addition of 12 mM succinate, followed by slope recording for 2 min. The reaction was terminated by addition of 15 mM malonate and CII activity was calculated as the malonate sensitive fraction, and expressed as nmol DCIP reduced.min^–1^.mg^–1^ protein.

The RC complex IV, cytochrome C oxidase, activity was measured by following the decrease in absorbance of reduced cytochrome C at 550 nm by spectrophotometry, as described by Barrientos et al. (2002). The assay was performed at +37°C using 15–20 μL of muscle homogenate in 1 mL of 10 mM potassium phosphate buffer pH 7.8 containing 1 mg.mL^–1^ BSA, 13.6 μM reduced cytochrome C, and 2.4 mM lauryl maltoside. The slopes were recorded for 1–2 min, stopped by addition of 100 μM KCN, and the results were expressed as nmol cytochrome C oxidized.min^–1^.mg^–1^ protein.

The activity of citrate synthase was determined by following the increase in absorbance of 5,5-dithio-bis-(2-nitrobenzoic acid) (DTNB) at 405 nm by spectrophotometry in 96-well microplates (Kamemura). The assay mixture (100 μL per well) contained 50 mM potassium phosphate buffer pH 7.8, 1 mM EDTA, 1 mg.ml^–1^ BSA, 100 μM DTNB and 200 μM acetylCoA. The reaction was initiated by addition of 5 μL diluted (1/2) muscle homogenate and reading for 2 min, after which 5 μL of 20 mM oxaloacetate were added for measurements of maximal activity. The results were expressed as nmol DTNB oxidized.min^–1^.mg^–1^ protein.

### mRNA Quantification by RT-qPCR Analysis

Twelve-month-old mice were anesthetized with intraperitoneal injection of pentobarbital 1% (6 μL.g^–1^). Perigonadic adipocytes were dissected and immediately frozen in liquid nitrogen. RNA was extracted using TRizol reagent (Invitrogen, Life Technologies, Saint-Aubin, France) with metal beads in 2 ml tubes and mechanically stressed with a TissueLyser apparatus (Qiagen). Each RNA preparation was treated with RQ1 RNase-Free DNase (Promega). One μg of mouse RNA was reverse transcribed with oligodT (20 mer) using reverse transcriptase Improm II (Promega France, Charbonnières, France). Quantitative real time PCR was performed with standard protocols using SYBR Green ROX as a fluorescent detection dye in ABI PRISM 7000 (ABgene, Courtaboeuf, France) in a final volume of 7 μL. Specific primers were used at 300 nM (Table 1). The cDNAs for the real time PCR were used at 5 ng/μL. The amounts of cDNA in each sample were determined on the basis of the threshold cycle (Ct) for each PCR product and normalized to proteasome 26 subunit [Rps 26 (26s)] Ct for all the tissues used. This housekeeping gene has been determined as best internal controls in our conditions, using Bestkeeper (Pfaffl et al., 2004) and Normfinder (Andersen et al., 2004) algorithms (data not shown). The calculated relative amount of mRNA was done respective to control samples and given as fold change after 2^–ΔΔ*CT*^ calculation (Livak method).

**TABLE 1:** RT-qPCR primers sequences.

**Gene**	**GenBank ID**	**Primer sequences**
**name**		
ACC1	NM_133360	Fw 5′-GCCTCTTCCTGACAAACGAG-3′ Rv 5′-TGACTGCCGAAACATCTCTG-3′
FASN	NM_007988	Fw 5′-AGAGATCCCGAGACGCTTCT-3′ Rv 5′-GCCTGGTAGGCATTCTGTAGT-3′
ACC2	NM_133904	Fw 5′-GAGCTGCTGTGTAAACACGAGATTGCT-3′ Rv 5′-CTGGTGCCGGCTGTCCTC-3′

### Statistical Analysis

All data are presented as mean and standard deviation (SD). For glucose and insulin tolerance test, as repeated measures experiments, a Holm–Sidak method was performed in order to compare two group of mice, while a two-way ANOVA test followed by *post hoc* Dunnet’s test was performed in order to compare multiple group of mice. For other multiple group comparisons, a Kruskal–Wallis test was performed followed by a *post hoc* Mann-Whitney test to verify significant differences between groups (GraphPad Prism v7.05, Chicago, IL, United States). For other two group comparisons, a non-parametric Mann–Whitney test was performed to verify significant differences (GraphPad Prism). All the data presented in this study were considered as statically different when the statistical power exceeds 95% (AnaStats.fr, France). All graphics were done with GraphPad Prism v7.05 and Adobe Illustrator CS6 v16.0.3.

## Results

### Low-Intensity Running and High-Intensity Swimming Enhanced Whole-Body Metabolism in SMA Mice

**FIGURE 1:**
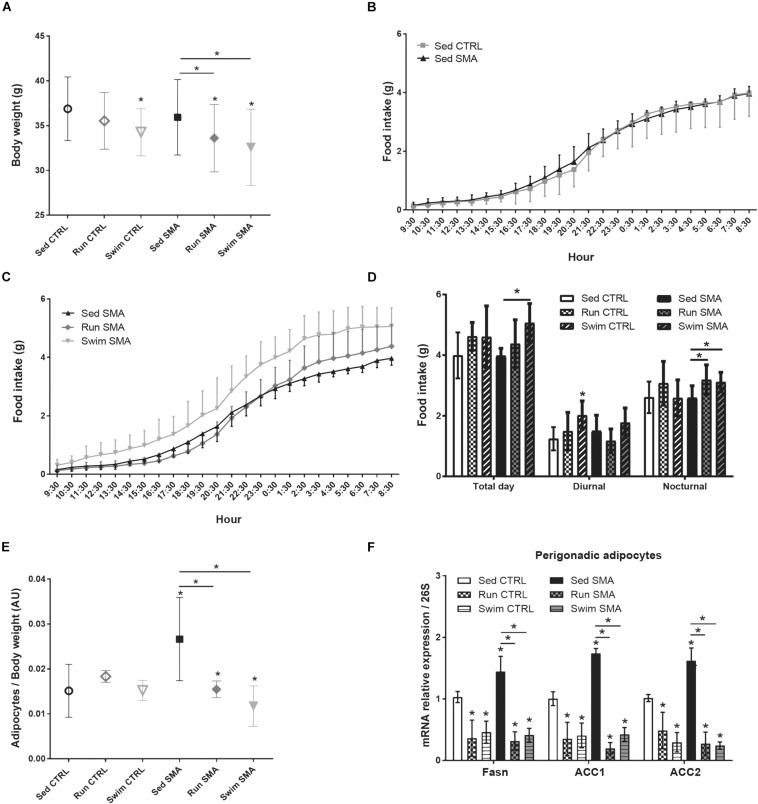
Whole body metabolism in sedentary and trained SMA and control mice. **(A)** Body weight of sedentary control (Sed CTRL *n* = 35), running-trained control (Run CTRL *n* = 18), swimming-trained control (Swim CTRL *n* = 18), sedentary SMA (Sed SMA *n* = 35), running-trained SMA (Run SMA *n* = 18) and swimming-trained SMA (Swim SMA *n* = 18) mice at 12 months of age. **(B,C)** 24 h food intake of **(B)** Sed CTRL (*n* = 12) compared to Sed SMA (*n* = 12) and **(C)** Sed SMA mice compared to Run (*n* = 8) and Swim (*n* = 6) SMA mice. **(D)** Quantification of food intake during all day (Total day), diurnal (9 h 30–21 h 30) and nocturnal periods (21 h 30–9 h 30) of sedentary and trained control and SMA mice at 12 months of age. **(E)** Ratio of white perigonadic adipocytes mass over body weight of sedentary and trained control and SMA mice at 12 months of age (*n* = 8 for each group). **(F)** Quantification of mRNA expression levels of *Fasn*, *Acc1* and *Acc2* by RT-qPCR in adipocytes of 12 months old sedentary and trained control and SMA mice (*n* = 4 for each group). ^∗^ and -^∗^- indicated significance relative to sedentary control and SMA mice, respectively (with *P* < 0.05).

The mild SMA model mouse, initially defined as a type III model mouse, has been well phenotypically characterized, with distal necrosis at 2 months of age (tail, ears and toes), quantifiable motor neurons degeneration at 6 months of age, a decrease in motor function and an increase in hind limb muscles atrophy from 9 months of age (Tsai et al., 2006; Chali et al., 2016). However, its metabolic status has never been investigated. Interestingly, despite the hind limb muscle atrophy, no significant modulation of the C57/B6 SMA body weight has been reported at any age when compared to age-matched controls (Tsai et al., 2006). We confirmed these data at 12 months of age in an FVB/NRj genetic background (Figure 1A). Interestingly, and for the first time, when we subjected SMA mice to 10 months low-intensity running or high-intensity swimming, we observed a significant reduction in body weight with both protocols when compared to sedentary SMA mice (Figure 1A). However, in control mice, unlike running, only swimming-based training significantly decreased the body weight at 12 months of age (Figure 1A), suggesting that the high-intensity swimming protocol induced higher effects than low-intensity running on body weight.

In order to determine whether this impact on body weight could be related to a modulation in food intake or to a metabolic change, we focused on the eating behavior of 12-month-old sedentary and trained mice during the whole day, i.e., the diurnal and nocturnal periods. In line with their lack of changes in body weight, the sedentary SMA and CTRL mice exhibited similar food (Figures 1B,D) and water (Supplementary Figures S1A,C) intakes, whatever the analyzed period of the day. Interestingly, in trained SMA mice, while both types of exercise significantly reduced the body weight, we observed an increase in food (Figures 1C,D) and water (Supplementary Figures S1B,C) intakes during the nocturnal phase when compared to sedentary SMA mice, without any significant modification during the diurnal phase. Moreover, in trained CTRL mice, a tendency to increase food (Figure 1D) and water (Supplementary Figure S1C) intakes had been observed for both protocols compared to sedentary CTRL mice, despite a swimming-induced significant increase in food intake during diurnal phase (Figure 1D). Taken as a whole, our data suggest that despite no modification in body weight and food intake in sedentary mice, both exercises induced whole-body metabolic change in CTRL and SMA mice by increasing nutrients intake and decreasing body weight.

Since the decrease in body weight observed in trained SMA mice was not linked to a decrease in food intake, we next investigated whether the modulation of body weight was related to a change in the adipose mass. We observed that the perigonadic white adipocytes mass reported on body weight was significantly greater in sedentary SMA mice, when compared to sedentary CTRL mice (Figure 1E). However, in trained SMA mice, both types of exercise totally restored this ratio, suggesting that running and swimming-based trainings in SMA mice could reduce the body weight by reducing adipose mass (Figure 1E). This ratio was not affected by any type of exercise in trained CTRL mice.

In order to determine whether the sedentary SMA-specific increase in adipose mass was a consequence of an impaired fatty acid metabolism, we focused on the gene expression of lipogenesis enzymes, *Fatty acid synthase* (*Fasn*), *Acetyl-CoA carboxylase 1* (*Acc1*) and *Acetyl-CoA carboxylase 2* (*Acc2*) in perigonadic white adipocytes of 12-month-old mice. Interestingly, the gene expression of *Fasn*, *Acc1* and *Acc2* was significantly increased in sedentary SMA mice, when compared to sedentary CTRL mice (Figure 1F). After exposing to the running and swimming protocols, we observed a significant reduction in the gene expression of all three enzymes in trained CTRL and SMA when compared to sedentary mice (Figure 1F). Thus, we showed that SMA induced lipogenesis which was counteracted by both exercise protocols.

Taken together, these results strongly suggest that the SMA mice exhibited an impaired fatty acid metabolism, which promotes lipogenesis without any body weight or food intake perturbations. Interestingly, both types of exercises reduce lipogenesis and decrease body weight with no change in food intake, whilst the swimming protocol provided more significant effects.

### Low-Intensity Running and High-Intensity Swimming Mitigate Glucose Homeostasis Alteration in SMA Mice

It is now well reported that alterations in body composition, lipogenesis and body weight can be related to glucose homeostasis defects, as observed in type 2 diabetes (Iizuka and Horikawa, 2008; Samuel, 2011; Samuel and Shulman, 2016). In addition, metabolism abnormalities such as metabolic acidosis, altered fatty acid metabolism, hyperlipidemia and hyperglycemia have been reported in SMA patients and mouse models (Dahl and Peters, 1975; Tein et al., 1995; Bowerman et al., 2012). However, no data are available in our SMA mouse model or on the metabolic effects of exercise in the SMA condition. To this purpose, we decided to evaluate glucose homeostasis in sedentary and trained CTRL and SMA mice from 3 to 12 months of age.

**FIGURE 2:**
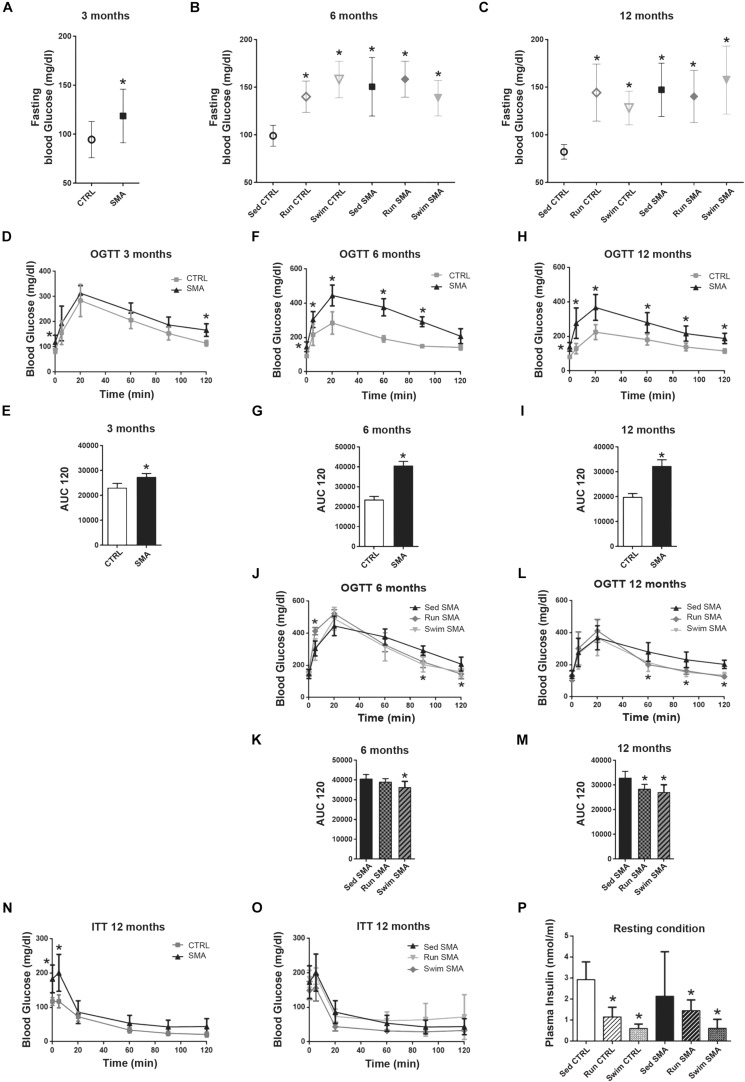
Glucose homeostasis in sedentary and trained SMA and control mice. **(A–C)** Fasting blood glucose measurement of **(A)** 3 months old sedentary control (CTRL) and SMA (SMA) mice, **(B)** 6 months old and **(C)** 12 months old sedentary and trained control and SMA mice. **(D–I)** Oral Glucose Tolerance Test (OGTT) and area under the curve (AUC) calculated over 120 min of **(D,E)** 3 months old, **(F,G)** 6 months old and **(H,I)** 12 months old Sed CTRL and Sed SMA mice (*n* = 8 for each group). **(J–M)** OGTT and area under the curve (AUC) calculated over 120 min of **(J,K)** 6 months old or **(L,M)** 12 months old Sed SMA compared to Run SMA and Swim SMA mice. **(N,O)** Insuline Tolerence Test (ITT) of **(N)** sedentary control and SMA mice and **(O)** Sed SMA compared to Run SMA and Swim SMA mice at 12 months of age (*n* = 8 for sedentary mice; *n* = 6 for trained mice). **(P)** ELISA quantification of plasma insulin levels of fed sedentary and trained controls and SMA mice at 12 months of age (*n* = 6 for each group). ^∗^Indicated significance relative to sedentary mice (with *P* < 0.05).

We found that sedentary SMA mice exhibited an early onset significant increase in fasting blood glucose, from 3 months of age, which increased to 6 months and then made a plateau to 12 months of age, when compared to sedentary CTRL mice (Figures 2A–C). Fasting hyperglycemia was maintained in trained SMA mice aged 6 and 12 months compared to sedentary SMA mice (Figures 2B,C). This hyperglycemia was also observed in trained CTRL mice when compared to sedentary CTRL mice at the same ages (Figures 2B,C), suggesting long-term exercise-specific adaptation. Moreover, sedentary SMA mice also exhibited glucose intolerance from the age of 3 months (Figures 2D,E), which increased to 6 months and reached a plateau to 12 months (Figures 2F–I) with significantly higher glucose levels and *Area under the curve* (AUC) following the oral glucose load, when compared to sedentary CTRL mice. However, both exercise protocols improved glucose tolerance by decreasing glucose levels following the oral glucose load at 90 and 120 min in 6-month-old SMA mice (Figures 2J,K), and at 60, 90, and 120 min in 12-month-old SMA mice (Figures 2L,M). Our results confirm that, also in our model mouse, glucose homeostasis is altered in SMA and suggest that both exercises modified glucose homeostasis by increasing glucose tolerance, despite maintenance of high glucose level in fasting condition.

We next questioned whether the hyperglycemia and glucose intolerance observed in sedentary SMA mice could be attributed to a reduced insulin sensitivity. To address this question, we performed an insulin tolerance test (ITT) in CTRL and SMA mice at 3 (Supplementary Figure S2A), 6 (Supplementary Figure S2B) and 12-month-old mice (Figure 2N). Interestingly, whatever the age, 20 min after insulin injection, the sedentary SMA mice did not displayed any blood glucose differences compared to sedentary CTRL mice, effects which were maintained until 120 min (Supplementary Figures S2A,B and Figure 2N). After both exercise protocols, we did not observed a modification of the insulin tolerance compared to sedentary SMA mice at 6 (Supplementary Figure S2C) and 12 months of age (Figure 2O).

In absence of impaired insulin sensitivity in sedentary SMA mice, we asked whether hyperglycemia and glucose intolerance could be due to an impaired insulin secretion. As already observed in other SMA mouse models (Bowerman et al., 2012, 2014), no significant difference in plasma insulin level was found between sedentary SMA and CTRL fed mice (Figure 2P) at 12 months of age, despite high variability in SMA. Interestingly, if both types of exercise did not significantly modify plasma insulin levels in the fed SMA mice, we observed a tendency to decrease plasma level with a decrease in the variability. We also observed a significant decrease in plasma insulin levels in trained CTRL mice (Figure 2P), suggesting that both protocols induced a decrease in insulin production in the fed mice.

Taken together, our results suggested that the sedentary SMA mice exhibit an abnormal glucose homeostasis, which seems largely insulin independent, and whose glucose intolerance was limited by both exercise protocols through a potentiation of insulin response.

### Low-Intensity Running and High-Intensity Swimming Attenuated SMA-Induced Oxygen Consumption Defects

As previously shown, SMA mice are characterized by impaired lipid metabolism and glucose homeostasis, and by chronic elevation of blood lactate in resting condition (Chali et al., 2016), which can be ameliorated by both exercise protocols (Chali et al., 2016). These data suggest a major abnormality in carbohydrate oxidation and energy expenditure in sedentary mice, which could be prevented by both exercise protocols. To address these issues, we investigated the systemic metabolism in 12-month-old sedentary and trained CTRL and SMA mice by indirect calorimetry with simultaneous measurements of total spontaneous activity.

**FIGURE 3:**
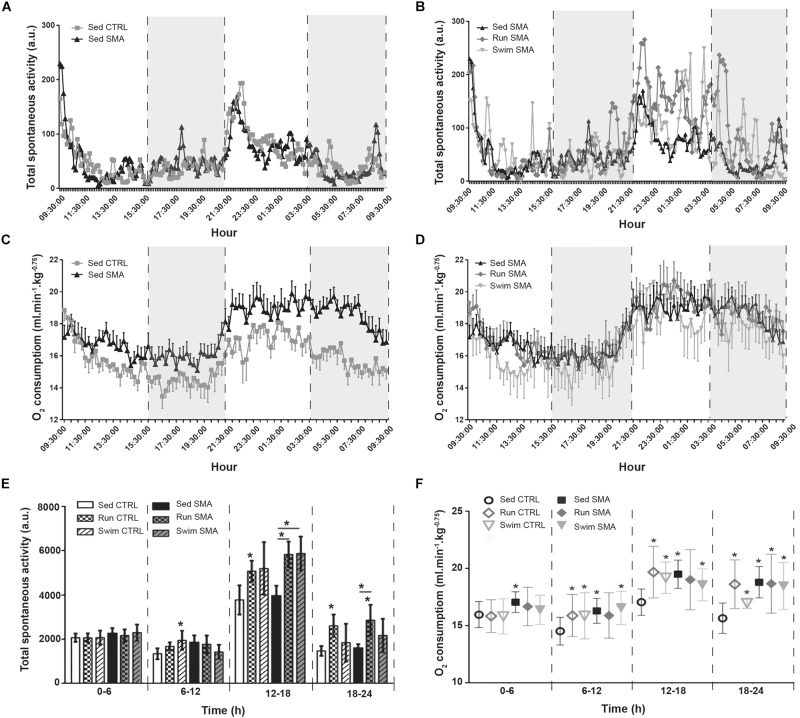
Oxymetry and spontaneous activity analysis in sedentary and trained SMA and control mice: **(A,B)** 24 h spontaneous activity of **(A)** sedentary control and SMA mice and **(B)** Sed SMA compared to Run SMA and Swim SMA mice at 12 months of age. Black dotted lines and gray background delimit periods of 6 h (9 h 30–15 h 30, 15 h 30–21 h 30, 21 h 30–3 h 30 and 3 h 30–9 h 30). **(C,D)** 24 h dioxygen consumption of **(C)** sedentary control and SMA mice, and **(D)** Sed SMA compared to Run SMA and Swim SMA mice at 12 months of age. **(E,F)** Quantification of spontaneous activity **(E)** and oxygen consumption **(F)** according to periods of 6 h from 12 months old sedentary and trained control and SMA mice (*n* = 12 for sedentary mice, *n* = 12 for Run CTRL, *n* = 8 for Run SMA and *n* = 6 for Swim mice). ^∗^ and -^∗^- indicated significance relative to sedentary control and SMA mice, respectively (with *P* < 0.05).

In sedentary CTRL and SMA mice, the total spontaneous activity appeared identical and followed the same circadian pattern, a low activity during the diurnal phase (9:30 am to 9:30 pm) and a nocturnal phase divided in two sub phases, with a high activity during the first one (9:30 pm – 3:30 am) and a low activity during the second one (3:30 am – 9:30 am) (Figure 3A). Surprisingly, the O_2_ consumption of sedentary SMA mice appeared higher than that of sedentary CTRL mice over 24 h, with a much greater difference during the second half of the nocturnal phase (Figure 3C). Thus, we decided to refine the analyses of each parameters by dividing the day in 4 periods of 6 h: two periods (0–6 h and 6–12 h) during the diurnal phase and two periods (12–18 h and 18–24 h) during the nocturnal phase.

We confirmed that sedentary SMA mice exhibited the same levels of spontaneous activity compared to the sedentary CTRL mice, whatever the analyzed period, with an important increase in activity for both SMA and CTRL mice during the 12–18 h period compared to the other periods (Figure 3E). We also confirmed that sedentary SMA mice exhibited significant higher O_2_ consumption compared to the sedentary CTRL mice, whatever the analyzed period (Figure 3F). However, during the nocturnal 18-24h period, the post-active period, we noted a twofold increase in O_2_ consumption difference between sedentary SMA and CTRL mice, suggesting defect in post-active recovery in SMA mice at 12 months of age (Figure 3F).

In trained SMA mice, both running and swimming exercises increased total spontaneous activity during the nocturnal phase, when compared to sedentary SMA mice (Figures 3B,D), without any modification of the O_2_ consumption (Figure 3F). While this spontaneous activity increase was significant for running SMA mice at both nocturnal periods, swimming exercise increased significantly this activity for the 12–18h period, but we observed just a tendency during 18–24 h period, when compared to sedentary SMA mice (Figure 3E). This suggest a restoration of the ratio activity/O_2_ consumption for trained mice and a decrease in the post-active overconsumption of O_2_. Interestingly, for control mice, both training protocols induced an elevation of oxygen consumption with the spontaneous activity increasing too, maintaining an energetic adequacy on all analyzed periods (Figures 3E,F).

Taken as a whole, our data suggest a drastic imbalance between O_2_ consumption and spontaneous activity in sedentary SMA mice compared to controls, which was worsened during the low activity period of the nocturnal phase, just after the high active nocturnal phase. This supports the hypothesis of a longer period of Excess Post-Exercise Oxygen Consumption (EPOC) (Borsheim and Bahr, 2003) in sedentary SMA mice. Importantly, both exercise protocols seemed to limit this discrepancy by restoring a normal ratio between O_2_ consumption and activity.

### Exercise-Specific Modulation of Nutrients Oxidation in SMA Mice

In order to evaluate if the observed systemic lipid and glucose homeostasis defects could parallel or inflict the discrepancy between O_2_ consumption and muscle activity, we focused on CO_2_ production to determine the RER and then the Fat and Carbohydrate (CH) oxidation levels in sedentary and trained control and SMA mice.

**FIGURE 4:**
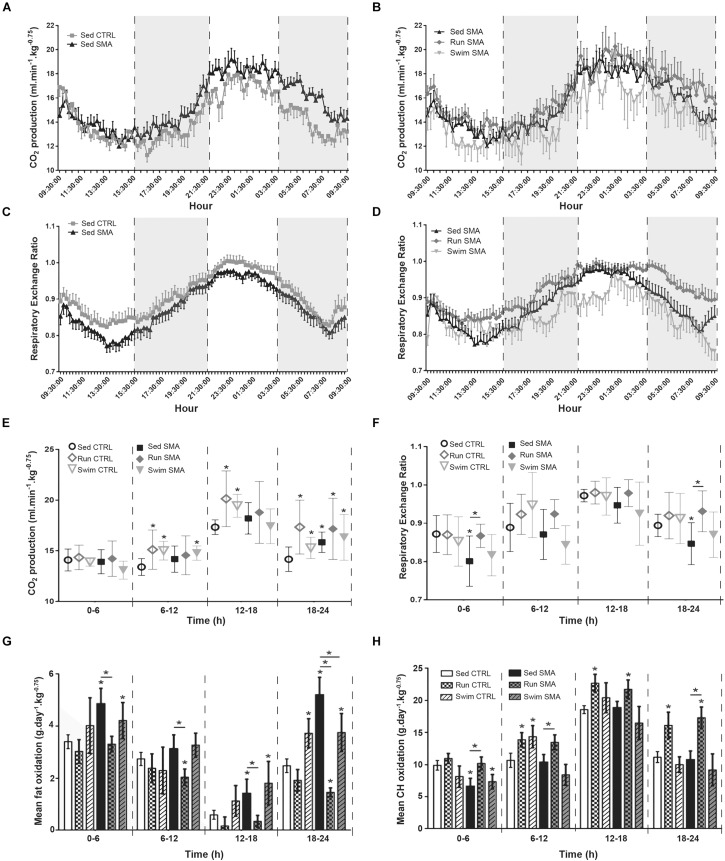
Nutrients oxidation measurements in sedentary and trained SMA and control mice. **(A,B)** 24 h carbon dioxide production of **(A)** sedentary control and SMA mice and **(B)** Sed SMA compared to Run SMA and Swim SMA mice at 12 months of age. Black dotted lines and gray background delimit periods of 6 h (9 h 30–15 h 30, 15 h 30–21 h 30, 21 h 30–3 h 30 and 3 h 30–9 h30). **(C,D)** 24 h respiratory exchange ratio of **(C)** sedentary control and SMA mice, and **(D)** Sed SMA compared to Run SMA and Swim SMA mice at 12 months of age. **(E,F)** Quantification of carbon dioxide production **(E)** and respiratory exchange ratio **(F)** according to periods of 6 h from 12 months old sedentary and trained control and SMA mice. **(G,H)** Quantification of mean fat oxidation **(G)** or carbohydrate oxidation **(H)** according to periods of 6 h from 12 months old sedentary and trained control and SMA mice (*n* = 12 for sedentary mice, *n* = 12 for Run CTRL, *n* = 8 for Run SMA and *n* = 6 for Swim mice). ^∗^ and -^∗^- Indicated significance relative to sedentary control and SMA mice, respectively (with *P* < 0.05).

In sedentary SMA mice, and in contrast with O_2_ consumption, the CO_2_ production was not significantly increased, except during the second half of the nocturnal phase (Figures 4A,E) and with lower amplitude when compared to O_2_ overconsumption (+8% of CO_2_ production and +24% of O_2_ consumption for sedentary SMA compared to CTRL mice). This led to a global shift toward a lower RER, indicating an increase in β-oxidation in sedentary SMA mice compared to CTRL mice at 12 months of age (Figures 4C,F), except during the high active 12–18 h period (Figure 4F). This metabolic shift was confirmed with a significant increase in total fat oxidation during all resting periods (0–6, 12–18, and 18–24 h), when compared to sedentary CTRL mice, and even worsening during the 18–24 h recovery period (Figure 4G). In contrast, except during the 0–6 h period, no significant modification of CH oxidation was observed in sedentary SMA mice compared to controls (Figure 4H). This confirmed the systemic metabolism alterations in sedentary SMA mice with a predominance of β-oxidation during the resting periods of the day.

Interestingly, if both exercise protocols did not significantly modify CO_2_ production in trained SMA mice when compared to sedentary SMA mice (Figures 4B,E), an important variability was observed for the low-intensity running protocol (Figure 4E). This induced a significant increase in RER (Figures 4D,F) in the low active periods (0–6 and 18–24 h), which was confirmed by a significant decrease in fat oxidation (Figure 4G) and an associated increase in CH oxidation (Figure 4H) when compared to sedentary SMA mice. In contrast, the high intensity swimming protocol did not modify significantly the RER (Figures 4D,F), neither fat (Figure 4G) nor CH (Figure 4H) oxidations during the entire 24 h of recordings when compared to sedentary SMA mice, but reduced fat oxidation during the recovery period (18–24 h). However, for the trained control mice, both protocols induced a significant increase in CO_2_ production from 6 to 24 h of recording (Figure 4E), which paralleled the increase in activity and O_2_ consumption when compared to sedentary controls (Figures 3E,F). So, we did not observe any significant shift in RER, only a slight increase (Figure 4F). Regarding nutrients oxidations, we confirmed that the low-intensity running protocol favored CH oxidation in trained control mice when compared to sedentary control mice, from 6 to 24 h, while the high-intensity swimming protocol favored fat oxidation, which was significant during the recovery period (18–24 h) (Figures 4G,H).

Altogether, these results showed higher fat oxidation levels in sedentary SMA mice when compared to control mice, which was exacerbated during the recovery period, supporting again the hypothesis of a longer period of EPOC in SMA. Importantly, we demonstrated that both exercise protocols were beneficial via exercise-specific modulation in nutrients oxidation, more prominent in SMA compared to controls, which supports the association between low-intensity running and CH oxidation, and between high-intensity swimming and fat oxidation.

### Low-Intensity Running and High-Intensity Swimming Reduced Energy Expenditure Defects in SMA-Mice

**FIGURE 5:**
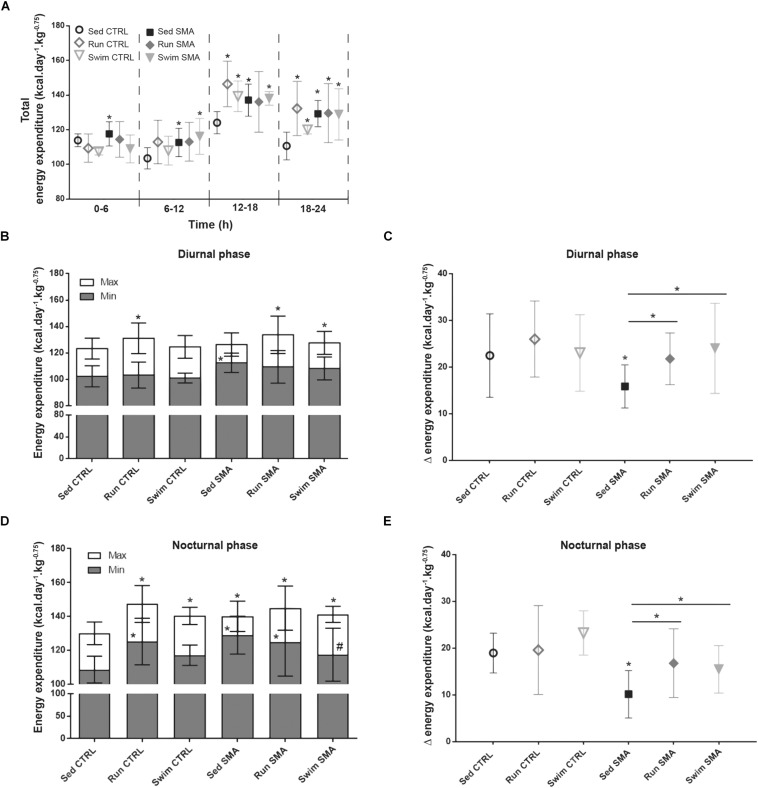
Energy expenditure in sedentary and trained SMA and control mice. **(A)** Quantification of total energy expenditure according to periods of 6 h from 12 months old sedentary and trained control and SMA mice. **(B,D)** Quantification of minimum (<10, Min) and maximum (>100, Max) activity energy expenditure in diurnal **(B)** and nocturnal **(D)** phases of 12 months old sedentary and trained control and SMA mice. **(C,E)** Quantification of the difference between minimum and maximum activity energy expenditure (D) in diurnal **(C)** and nocturnal **(E)** phases from 12 months old sedentary and trained control and SMA mice (*n* = 12 for sedentary mice, *n* = 12 for Run CTRL, *n* = 8 for Run SMA and *n* = 6 for Swim mice). ^∗^ and -^∗^- Indicated significance relative to sedentary control and SMA mice, respectively (with *P* < 0.05).

In sedentary SMA mice, the global alteration in oxidative metabolism with an uncoupling between O_2_ consumption and muscular activity led us to hypothesize about a possible enhancement in basal EE and so, a reduction in energy supply. In order to test this hypothesis and determine if exercise protocols could reduce metabolic alterations, we evaluated total energy expenditure (EE) during the four periods for the sedentary and trained control and SMA mice at 12 months of age (Figure 5A). Moreover, we refined this analysis by evaluating mice EE during either resting condition (activity below 10, Min) or active condition (activity up to 100, Max) in diurnal and nocturnal phases, pointing out the delta of EE between minimal and maximal EE.

Concerning total EE, sedentary SMA mice showed a significant increase compared to sedentary control mice, whatever the analyzed period (Figure 5A). This increase in EE could not be explained by a defect in thermoregulation since sedentary SMA and CTRL mice presented the same rectal temperature during ambient air, cold air and cold water stress tests (Supplementary Figures S3A,B). However, during the nocturnal 18–24 h period, the post-active period, we noted that the difference in total EE between sedentary SMA and CTRL mice increased twofold (<+10% of EE between 0 and 18 h for >+20% between 18 and 24 h), confirming the persistence of high EE during the low active phase and long after the high active phase (Figure 5A). This alteration was also observed by comparing the EE during minimum activity and the EE during maximum activity. Interestingly, SMA mice showed significant increase in minimal EE for both diurnal and nocturnal phases (Figures 5B,D) which induced a decrease in the delta of EE for the same periods (Figures 5C,E). Taken together, these results suggest a decrease in energy supply in sedentary SMA mice due to an increase in resting EE, limiting their ability to adapt their energy to further elevation in activity.

If both exercise protocols did not modify the total EE in trained SMA mice compared to sedentary SMA mice (Figure 5A), both protocols significantly enhanced the delta of EE for both diurnal and nocturnal phases (Figures 5C,E). For the running exercise, this energy supply enhancement was due to a non-significant elevation of maximal EE combined with a non-significant decrease in minimal EE. In contrast, for the swimming exercise, this benefit mainly due to a reduction in minimal EE, only significant during nocturnal phase (Figures 5B,D). However, for the trained controls, we only observed a significant elevation of total EE during the nocturnal phase (Figure 5A). This increase could be associated with the significant increase in spontaneous activity (Figure 3E). Finally, training protocols did not alter the delta between minimal and maximal EE for both diurnal and nocturnal phases despite a tendency to increase in CTRL mice (Figures 5C,E).

Taken as a whole, our data support a specific alteration in basal EE for an equivalent activity, which reduces energy supply for sedentary SMA mice, and which points out alterations in muscular mitochondria function. However, despite protocol-specific adaptations, both exercise protocols seemed to limit this energy supply reduction, which raised the hypothesis of an exercise-induced enhancement of mitochondrial functions in SMA.

### Low-Intensity Running and High-Intensity Swimming Reduced Mitochondria Defects in SMA-Mice

Our systemic and whole-body evaluation of metabolic status in SMA mice pointed out specific alterations in oxidation processes and energy production, confirming muscular mitochondria alterations (Sperl et al., 1997; Berger et al., 2003; Ripolone et al., 2015) also in our mild SMA model mouse. With the elevation of resting EE and long-term O_2_ overconsumption post-active phase, we could hypothesize that the efficiency of muscle mitochondria to produce energy is decreased in SMA. To address these issues and to determine how physical exercises could induce metabolic benefits, we evaluated mitochondria status in three different muscles of the calf, the fast-twitch flexor *tibialis*, the fast-twitch extensor *plantaris* and the slow-twitch extensor *soleus*, in sedentary SMA and CTRL mice and in trained SMA mice at 12 months of age.

**FIGURE 6:**
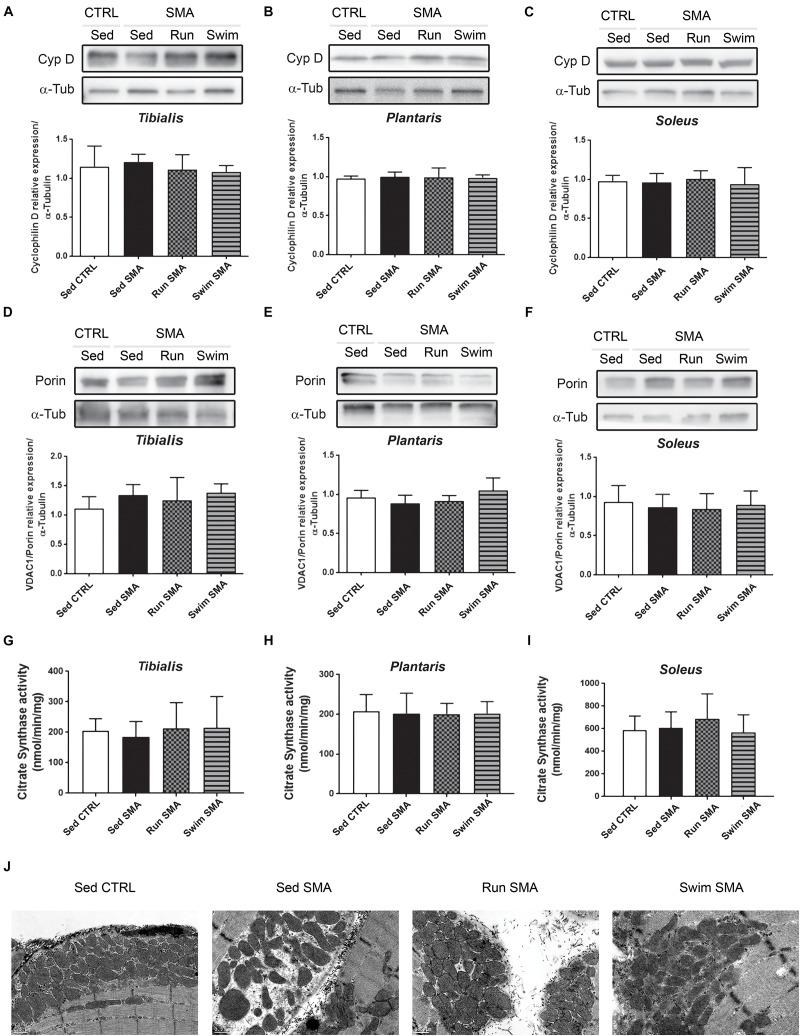
Muscular mitochondria quantification and qualification in sedentary and trained SMA mice. **(A–F)** Western blot analysis and quantification of cyclophilin D **(A–C)** and VDAC1/porin **(D–F)** steady state protein levels in **(A,D)** fast-twitch flexor *tibialis*, **(B,E)** fast-twitch extensor *plantaris* and **(C,F)** slow-twitch extensor *soleus* of sedentary control mice compared to sedentary and trained SMA mice (*n* = 4 for each group). **(G–I)** Quantification of citrate synthase activity in **(G)** *tibialis*, **(H)** *plantaris*, and **(I)** *soleus* of sedentary control mice compared to sedentary and trained SMA mice at 12 months of age (*n* = 6). **(J)** Transmission electron microscopy images of submembranar mitochondria in ultrathin (50–90 nm) *tibialis* muscles longitudinal sections from 12 months old sedentary control mice compared to sedentary and trained SMA mice (4000X magnification images).

We analyzed the total amount of mitochondria in sedentary SMA mice compared to control mice via three complementary approaches. Firstly, we quantified by western-blot the amount of Cyclophilin-D, part of the mitochondrial Permeability Transition Pore (PTP) and the outer mitochondrial membrane porin (also called VDAC1) which are usually used as markers of mitochondria enrichment (Webster et al., 2013). No significant differences were observed in the steady state proteins levels in sedentary SMA mice compared to controls (Figures 6A–F). Secondly, we measured the citrate synthase activity from the Krebs cycle of the mitochondria matrix and failed to observe any significant differences in activity between sedentary SMA and control mice (Figures 6G–I). Finally, no qualitative differences were observed by transmission electron microscopy on mitochondria amount and structure in *tibialis* (Figure 6J and Supplementary Figure S4) and *soleus* (Supplementary Figure S4) longitudinal sections. Our results suggest a preservation of the total amount of mitochondria in analyzed hind limb muscles of our SMA model mouse at 12 months of age. Interestingly, both exercise protocols failed to promote mitochondria biogenesis in SMA muscles, as observed by western blot (Figures 6A–F), enzymatic activity (Figures 6G–I) or by qualitative transmission electron microscopy (Figure 6J and Supplementary Figure S4) despite energetic benefits in whole-body measurements.

**FIGURE 7:**
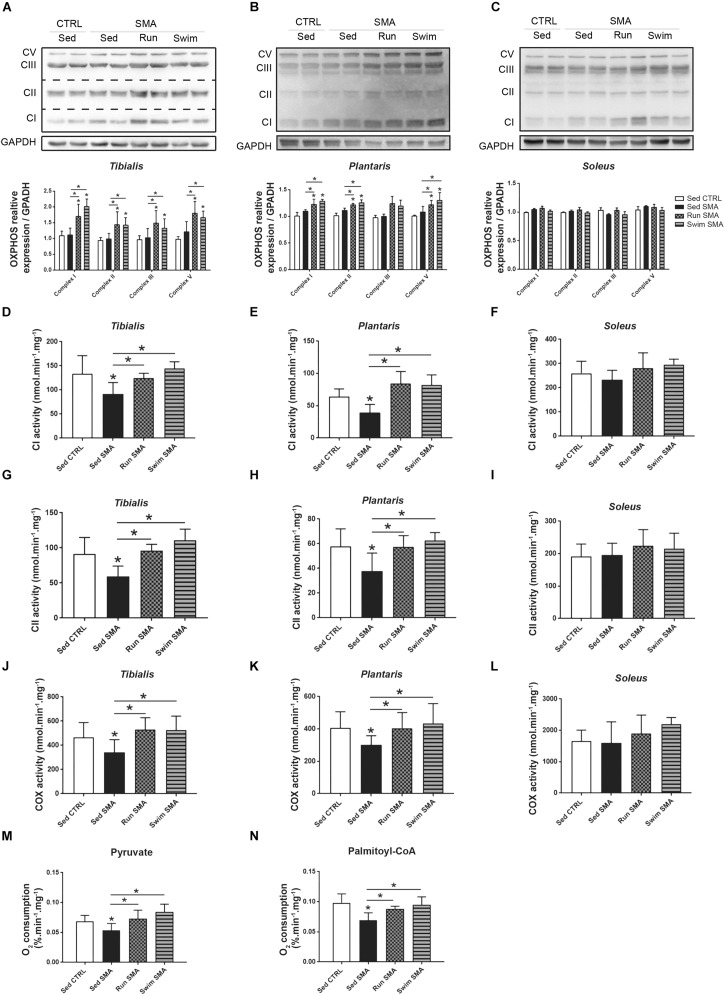
Muscular mitochondrial function in sedentary and trained SMA mice. **(A–C)** Western blot analysis and quantification of CV, CIII, CII, and CI respiratory chain complexes steady state protein levels in **(A)** *tibialis*, **(B)** *plantaris* and **(C)** *soleus* of sedentary control mice compared to sedentary and trained SMA mice (*n* = 4 for each group). **(D–L)** *In vitro* maximal enzymatic activity measurement of **(D–F)** CI, **(G–I)** CII, and **(J–L)** CIV (COX) respiratory chain complexes in **(D,G,J)** *tibialis*, **(E,H,K)** *plantaris* and **(F,I,L)** *soleus* of sedentary control mice compared to sedentary and trained SMA mice (*n* = 6 for each group). **(M,N)** *In vitro* dioxygen consumption measurement of *tibialis* and *plantaris* isolated mitochondria with pyruvate **(M)** and palmitoyl-CoA **(N)** substrates from sedentary control mice compared to sedentary and trained SMA mice (*n* = 6 for each group). ^∗^ and -^∗^- Indicated significance relative to sedentary control and SMA mice, respectively (with *P* < 0.05).

Taken together, these results suggest that metabolic alterations in SMA and beneficial effects of exercise protocols should rely on mitochondria function. This led us to evaluate the expression and enzymatic activity of the different respiratory chain complexes. Using OXPHOS mix antibody by western blot analysis, we did not observe difference in the steady state protein level of all respiratory complexes and for all analyzed muscles of sedentary SMA mice compared to controls (Figures 7A–C). However, using *in vitro* maximal enzymatic activity measurements of the respiratory chain complex I (NADH:ubiquinone oxidoreductase), complex II (succinate dehydrogenase) and complex IV (cytochrome c oxydase), we noted a significant reduction in their activity in *tibialis* and *plantaris* only (Figures 7D,E,G,H,J,K), while no significant modification was measured for the slow-twitch *soleus* muscle (Figures 7F,I,L). This result suggests a muscle-specific alteration in respiratory chain function, involving phasic fast-twitch muscles and sparing tonic slow-twitch muscle.

Interestingly, both exercise protocols promoted respiratory chain complexes expression in fast-twitch *tibialis* and *plantaris* muscles, but not in slow-twitch *soleus* (Figures 7A–C). This muscle-specific overexpression of complexes were associated with a restoration of enzymatic activity in fast-twitch muscles of SMA mice to sedentary control values (Figures 7D,E,G,H,J,K). No significant differences in complexes activity were observed in the slow-twitch *soleus* despite a tendency toward an increase (Figures 7F,I,L). So, exercise seemed to enhance mitochondrial function through an increased respiratory chain complexes overexpression.

Finally, we sought to understand the link between mitochondrial function alterations and the systemic ones, by evaluating their ability to consume O_2_ under either carbohydrate or lipid substrate. We performed mitochondrial isolation of both fast-twitch muscles and we measured their maximal rate of O_2_ consumption with either pyruvate or palmitoyl-CoA substrate, *in vitro*. Interestingly, we observed a decrease in O_2_ consumption for both substrates in sedentary SMA mice compared to control mice (Figures 7M,N), supporting a decrease in mitochondrial efficiency in producing energy. However, after both physical trainings, isolated mitochondria from fast-twitch muscles recovered their ability to consume O_2_ under both substrates (Figures 7M,N), with a tendency in swimming exerting better effects than running.

Taken together, these experimental results support the hypothesis of a muscle-specific alteration in mitochondrial efficiency, without changes in mitochondrial quantity or in the respiratory chain complexes expression, the latter being restored by both exercise protocols.

## Discussion

The high variability in metabolic alterations in SMA patients (Dahl and Peters, 1975; Bruce et al., 1995; Tein et al., 1995; Crawford et al., 1999; Berger et al., 2003; Sproule et al., 2009; Zolkipli et al., 2012; Davis et al., 2015; Bertoli et al., 2017; Kolbel et al., 2017) and mouse models (Bowerman et al., 2012, 2014; Ripolone et al., 2015) can preclude or limit the application, design or efficiency of therapies, including active exercise. Thus, deciphering the origin and mechanisms involved in those metabolic alterations and the way to modulate them appeared crucial. In the present work, we addressed two original points. One, we provide the first lines of evidence indicating mild SMA-like mice are affected by a state of “chronic oxygen debt” due to a decrease in fast-twitch SMA muscle mitochondrial efficiency, which might be at the root of SMA-induced metabolic alterations. This “chronic oxygen debt” in mild SMA-like mice induces elevation in resting O_2_ consumption and lipids oxidations, both worsening after long periods of activity. It also induces an elevation in basal EE, and the decrease in energy supply. This metabolic shift may ultimately inflict the systemic metabolic perturbations already reported in SMA mice and patients, including glucose homeostasis impairments and lipid overload. Two, long-term exercise protocols were able to significantly enhance mitochondrial efficiency in SMA fast-twitch muscles, promoting glucose re-use and enhancing lipid metabolism. However, we demonstrated that the high-intensity swimming benefits go through elevated β-oxidation while low-intensity running benefits go through carbohydrate oxidation, showing exercise-specific adaptations.

Usually, the concept of “oxygen debt” is used to characterize the metabolic state of skeletal muscles during physical exertion (Borsheim and Bahr, 2003). In that case, a cumulative deficit of oxygen results from the high energetic demand imposed to muscles to sustain increased workload during physical exercise. When muscles require more energy than oxidations can deliver, the anaerobic pathway compensates the deficit, resulting in lactate production, in the progressive decrease in strength and then fatigue. When the exercise stops, an excess postexercise oxygen consumption (EPOC) or reimbursement of the oxygen debt occurs, with the body consuming more oxygen than usual at rest for up to 24 h. This recovery period allows the elimination of the produced lactate, replenishment of energy substrate stocks, restoring fuel balance and allowing cellular adaptations through the β-oxidation pathway (Kiens and Richter, 1998; Egan and Zierath, 2013). In the SMA context, for which maximal mitochondria oxygen consumption is significantly reduced from both carbohydrate and lipid substrates (Figures 7M,N), the spontaneous activity of mice is sufficient to chronically induce an oxygen debt. The present results show for the first time, an abnormally elevated EE at rest in SMA mice (Figures 5B,D), independent of thermoregulation defects, mainly supported by β-oxidation pathway (Figures 4C,F,G) and worsening during the post-active period of the nocturnal phase (Figure 5A). These results are also highly consistent with previous data reporting a chronic elevation of resting lactatemia in SMA-like mice (Chali et al., 2016). Thus, with a reduced maximal mitochondria oxygen consumption, SMA induces a chronic overuse of oxidation at rest, compared to control mice (Figure 3C). A second consequence of this “chronic oxygen debt” is the reduction of muscular energy supply in SMA mice. The increase in basal EE, to reimburse oxygen debt and/or produce sufficient energy for basal behavior, reduces the delta of energy between rest and muscular activity, enhancing in turn the risk of “oxygen debt” when mice will be active. This process appears as a feed-forward negative loop. Finally, this feature of “chronic oxygen debt” and reduced mitochondria efficiency in SMA could explain, at least in part, the muscular fatigue commonly described in SMA patients (Montes et al., 2010; Pera et al., 2017).

Importantly, this state of “chronic oxygen debt” was limited by long-term physical exercise in mild SMA-like mice, but in an exercise-type manner. Both exercise protocols improved mitochondria oxygen consumption from both carbohydrate and lipid substrates, subsequently reducing the resting oxidation function and therefore increasing muscle energy supply (Figures 5C,E). However, the swimming-based training induced a decrease in the resting EE while running increases the maximal EE during the nocturnal phase. Thus, in both cases, we observed an increase in the amplitude of energy supply during the nocturnal active phase, allowing better adaptation to any further increased in workload (Figures 5B–E). In this study, the effect differences between the two exercise protocols may be related to the differential use of energetic substrates linked to exercise intensity (Chali et al., 2016). Indeed, while the swimming-based training maintained a strong use of the lipid β-oxidation, without modifying the use of carbohydrate, the running-based training shifted the metabolism from β-oxidation toward the use of carbohydrates oxidation (Figures 4G,H), as also suggested by the decreased lactatemia in the running-based trained mice (Chali et al., 2016). Interestingly, this exercise-induced difference in energy pathway could explain, by modulating the RER, the elevated resting EE in running-based trained SMA-like mice compared to swimming SMA-like mice, as observed in the trained control mice (Figures 5B,D).

Interestingly, we found that only the fast-twitch muscles were affected by these mitochondrial activity defects, suggesting that the state of “chronic oxygen debt” could mainly originate from the limitation of fast-twitch muscles to catabolize carbohydrates by the aerobic pathway. Furthermore, this muscle-specific defect is likely to explain the high lactatemia found in mild SMA-like mice. To sustain the energy demand during the active phase, the fast-twitch muscles may then use lipids as replacement substrates, but with limited energetic power since β-oxidation only occurs in aerobic conditions and with multiple enzymatic steps (Fillmore and Lopaschuk, 2013). The consequence of these muscular mitochondrial defects are reflected at systemic level, with a decrease in glucose use, highlighted by glucose intolerance, and an increase in lipid use, highlighted by the decrease in the RER in SMA-like mice. Consistently, both exercise induced significant increases in the expression of mitochondrial respiratory chain complexes in fast-twitch muscles, resulting in significant improvements in oxidative capacities of muscular SMA mitochondria. Interestingly, the oxidative activity, *per* respiratory chain complex, remained unchanged in exercised muscles compared to sedentary ones, suggesting that the increased oxidative efficiency induced by exercise relates to the increased number of respiratory chain complexes, and not to their intrinsic functional properties. Moreover, the number of mitochondria *per* muscle did not change. Therefore, improving the mitochondrial oxidative capacity could alone explain the beneficial effects of exercise. Indeed, with an aerobic metabolism functioning properly again, fast-twitch muscles can reuse glucose via the aerobic pathway, thus limiting lactate production and, finally, glucose resistance. Ultimately, the state of “chronic oxygen debt” is reduced, as is the resting EE.

Surprisingly, the slow-twitch *soleus* muscle seems spared from SMA-induced alterations in respiratory chain function compared to fast-twitch *tibialis* and *plantaris*. This muscle-specific metabolic alteration has never been reported yet and could be partly explained by two main physiological points. Firstly, slow-twitch muscles are characterized by high amount of mitochondria with an oxidative-based metabolism allowing them to efficiently catabolize lipids, while fast-twitch muscles favor carbohydrate catabolism. So, in the context of SMA with glucose resistance and hyperlipidemia, slow-twitch muscle should suffer less compared to fast-twitch muscles. Secondly, slow-twitch muscles are tonic, that mean continuously contracted to maintain posture against gravity, while fast-twitch muscles are phasic, contracting only for movement. Putting these muscle specificities in parallel with our data supporting the fact that the more active is the muscle, more its function is protected and efficient, could also explain this muscle-specific alteration. Thus, our data reinforce the hypothesis which link neuromuscular activity and protection against SMA (Chali et al., 2016).

Glucose homeostasis impairments have been already reported in other SMA mouse models (Bowerman et al., 2012, 2014). Consistently with these previous results, we report here a fasting hyperglycemia in mild SMA-like mice compared to controls. This defect occurs as early as 3 months, thus far away before the beginning of MN death in this model (Tsai et al., 2006). Interestingly, SMA tissues remained sensitive to insulin, suggesting a SMA-induced impairment in the equilibrium between blood insulin and glucagon concentrations, as previously shown (Bowerman et al., 2012, 2014). In trained SMA-like mice, although fasting glycemia remained high and unchanged compared to sedentary SMA-like mice, the glucose tolerance was significantly improved, as expected with exercise which improves directly insulin response in muscles (Way et al., 2016; Kleinert et al., 2018).

Moreover, the systemic and muscular metabolic impairments recorded in sedentary mild SMA-like mice, and their adaptations under exercise paradigms are also evidenced at the systemic level. Sedentary mild SMA-like mice displayed the same weight curve and the same levels of food intake as control mice but had yet more adipocytes. High lipid storage is expected in case of glucose resistance and preferential lipid use for insuring energetic metabolism (Samuel and Shulman, 2016). Consistently with these observations, the mRNA expression of lipogenic enzymes such as *Acc1*, *Acc2* and *Fasn* were found significantly increased in SMA fat tissues. Expectedly, both exercise protocols resulted in a significant decrease in fat mass and body weight, with yet a tendency to increase food intake in SMA-like mice. These benefits could be due to the enhancement in glucose tolerance and associated with a significant decrease in the mRNA expression of lipogenic enzymes in fat tissues, demonstrating a whole-body metabolism enhancement.

Finally, this study opened new avenues in the SMA pathophysiology decryption, pointing out the alteration in intrinsic respiratory chain function as a possible key for a large part of metabolic defects. Without any modification in the total amount of those crucial complexes, this specific decrease in maximum activity should rely to post-translational modifications, mechanism already reported in other neurodegenerative disorders (Nakamura and Lipton, 2017) and need to be deciphered in SMA via a dedicated research project.

Thus, taken together, these findings argue for the use of physical exercise as a therapeutic intervention for SMA patients, in complement to pharmacological or gene-therapies aimed at improving SMN expression in MNs. To do so, two key elements should be taken into account, the metabolic status and the fatigue.

It is admitted today that the metabolic status of SMA patients are diverse, notably in terms of diabetes, obesity or insulinemia (Davis et al., 2015; Kolbel et al., 2017), resulting in different abilities for them to perform active exercise. Therefore, particular attention should be paid to the exercise paradigm used, in order to fit the training protocol to the expected metabolic adaptation of each patient. In this way, it is important to note that the current study has been conducted only on male mice, in accordance with the original work we recently published on the neuroprotective effects of physical exercise in type 3 SMA-like mice (Chali et al., 2016). Nevertheless, it is now well described that the gender can inflict both the metabolic status (Medrikova et al., 2012; Venezia et al., 2016; Reynolds et al., 2019) and the exercise-induced benefits (Veldink et al., 2003; McMullan et al., 2016; Zhou et al., 2018). Thus, it seems crucial to evaluate if female SMA mice with the same genetic background than the males used in the present study could identically adapt to the same exercise protocols.

Finally, the muscular fatigue appears as an important feature in SMA. For instance, the main complaint of type 3 SMA patients during the application of a 12-week aerobic training on ergo cyclometer was an excessive fatigue, even if exercise intensity was low (Madsen et al., 2015). So, the application of an intensive active physiotherapy could appear contradictory. Nonetheless, we demonstrated here that high-intensity exercise i.e., the swimming-based protocol, expected to induce high fatigue levels, has highest beneficial effects than low-intensity exercise i.e., the running-based protocol. These results suggest that a threshold of exercise intensity, and therefore of fatigue, should be exceeded repeatedly throughout the training, in order to obtain beneficial effects on muscular function. Thus, if we take into account the two studies (Madsen et al., 2015 and the present study), a high-intensity exercise would be necessary, even if it induces a high level of fatigue during exercise, to improve muscle function metabolism, ultimately improving resistance to current life fatigue.

All those elements, taken as a whole, support the necessity to use precision and personalized intervention in order to optimize exercise-induced benefits for SMA patient care.

## Data Availability Statement

All datasets generated for this study are included in the manuscript/Supplementary Files.

## Ethics Statement

Animal handling and experimentation were performed in line with approved Institutional Animal Care and Use Committee protocols at the University of Paris Descartes (CEEA 34, agreement number B75-06-07) and followed the national authority (Ministere de la Recherche et de la Technologie, France) guidelines for the detention, use and the ethical treatment of laboratory animals based on European Union Directive 2010/63/EU.

## Author Contributions

LH conducted and analyzed the majority of experiments. DD’A conducted the mRNA, adipocytes, and glucose homeostasis experiments. JB supervised, participated, and analyzed the mitochondrial experiments. FaC and CD participated in the animal care, glucose homeostasis, and tissues collection. VR conducted and analyzed the Western blot experiments. JS and CO participated and analyzed the enzymatic activity measurements. TR and BB conducted and analyzed the physiocages experiments and participated in the writing of the manuscript. JR supervised the physiocages experiments. DS helped in animal care and tissues collection. LW and PL helped in the writing of the manuscript. FD helped in analyzing the data and writing of the manuscript. CB participated in the mRNA experiments and animal care. FrC helped in supervising the project and writing of the manuscript. OB supervised the project, conducted the mitochondrial experiments, analyzed the data, and wrote the manuscript. All authors have approved the final version of the manuscript and agreed to be accountable for all aspects of the work.

## Conflict of Interest

CB was employed by company Biophytis. TR, BB, and JR were employed by company Biomeostasis. A patent on active physiotherapy for SMA patients is pending. The remaining authors declare that the research was conducted in the absence of any commercial or financial relationships that could be construed as a potential conflict of interest.
