# Supplementary material for: Low-Intensity Running and High-Intensity Swimming Exercises Differentially Improve Energy Metabolism in Mice With Mild Spinal Muscular Atrophy
Source: Front Physiol. 2019 Oct 1;10:1258. doi: 10.3389/fphys.2019.01258 (PMC6781613; doi:10.3389/fphys.2019.01258)
Supplement: Supplementary file 1 [file Data_Sheet_1.PDF]

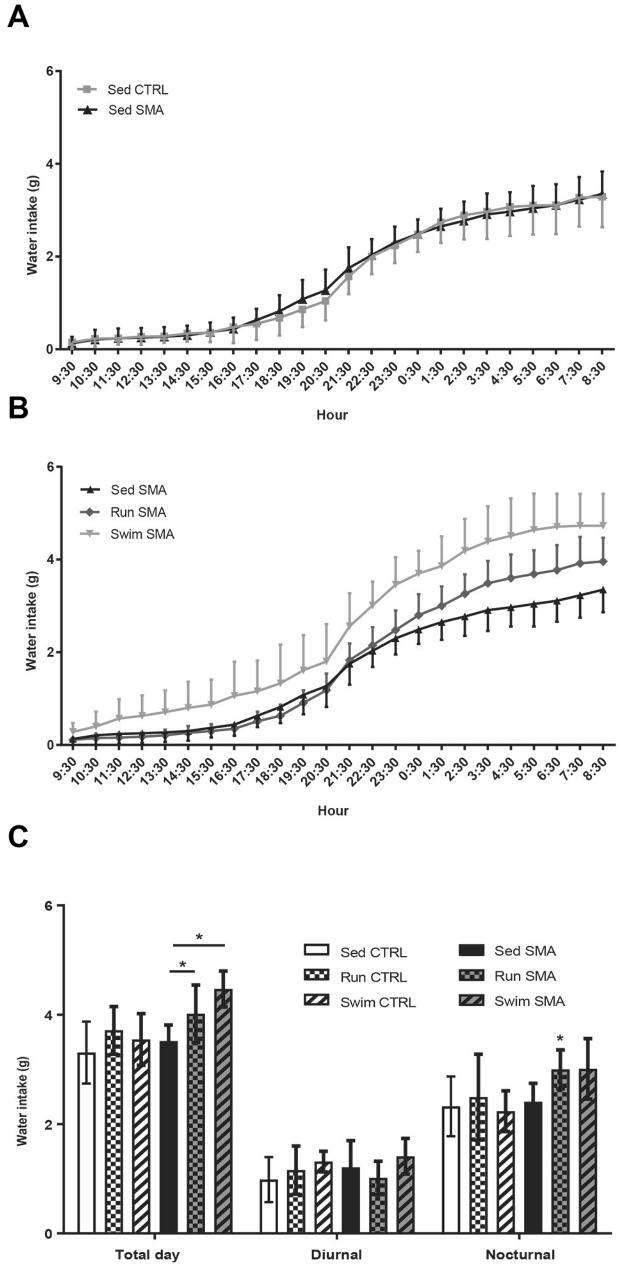

Supplementary Figure S1. *Water intake in sedentary and trained SMA and control mice.* (**A**) (**A, B**) 24h water intake of (**A**) sedentary control mice (Sed CTRL, n=12) compared to sedentary SMA mice (Sed SMA, n=12) and (**B**) Sed SMA mice compared to running-trained SMA mice (Run SMA, n=8) and swimming-trained SMA mice (Swim SMA, n=6) at 12 months of age. (**C**) Quantification of water intake during all day (Total day), diurnal (9h30-21h30) and nocturnal periods (21h30-9h30) of sedentary and trained control and SMA mice at 12 months of age. * and -*- indicated significance relative to sedentary control and SMA mice, respectively (with $P<0.05$).

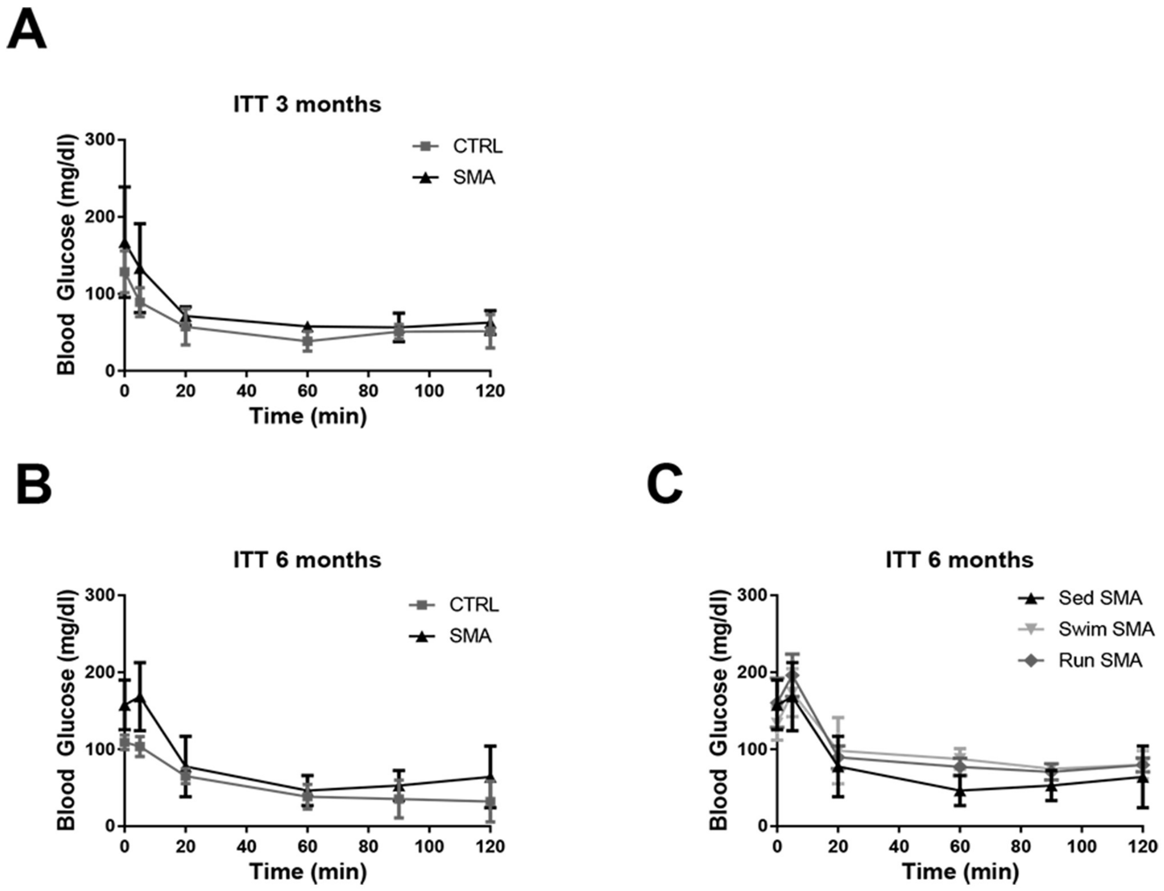

Supplementary Figure S2. *Insulin tolerance test in sedentary and trained SMA mice*. (**A-C**) Insuline Tolerence Test (ITT) of **(A)** 3 months old, (**B**) 6 months old sedentary control (Sed CTRL) and SMA (Sed SMA) mice and **(C)** 6 months old Sed SMA compared to Run SMA and Swim SMA mice (n=8 for sedentary mice; n=6 for trained mice). * indicated significance relative to sedentary mice (with P<0.05).

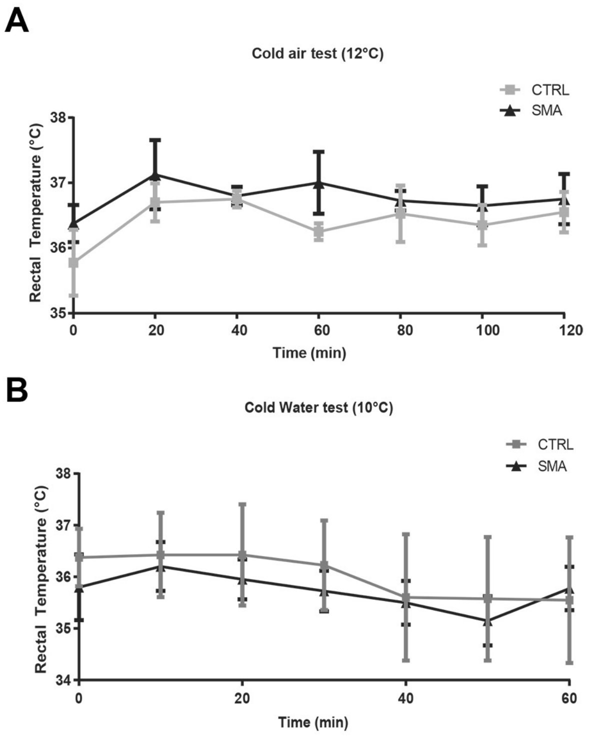

Supplementary Figure S3. *Thermoregulation in sedentary control and SMA mice*. (**A-B**) Rectal temperature measurements of sedentary control (Sed CTRL) and SMA (Sed SMA) mice at 12 months of age subjected to **(A)** water cold, for 60 minutes, and (**B**) air cold, for 120 minutes, stress tests (n=4 for each group).

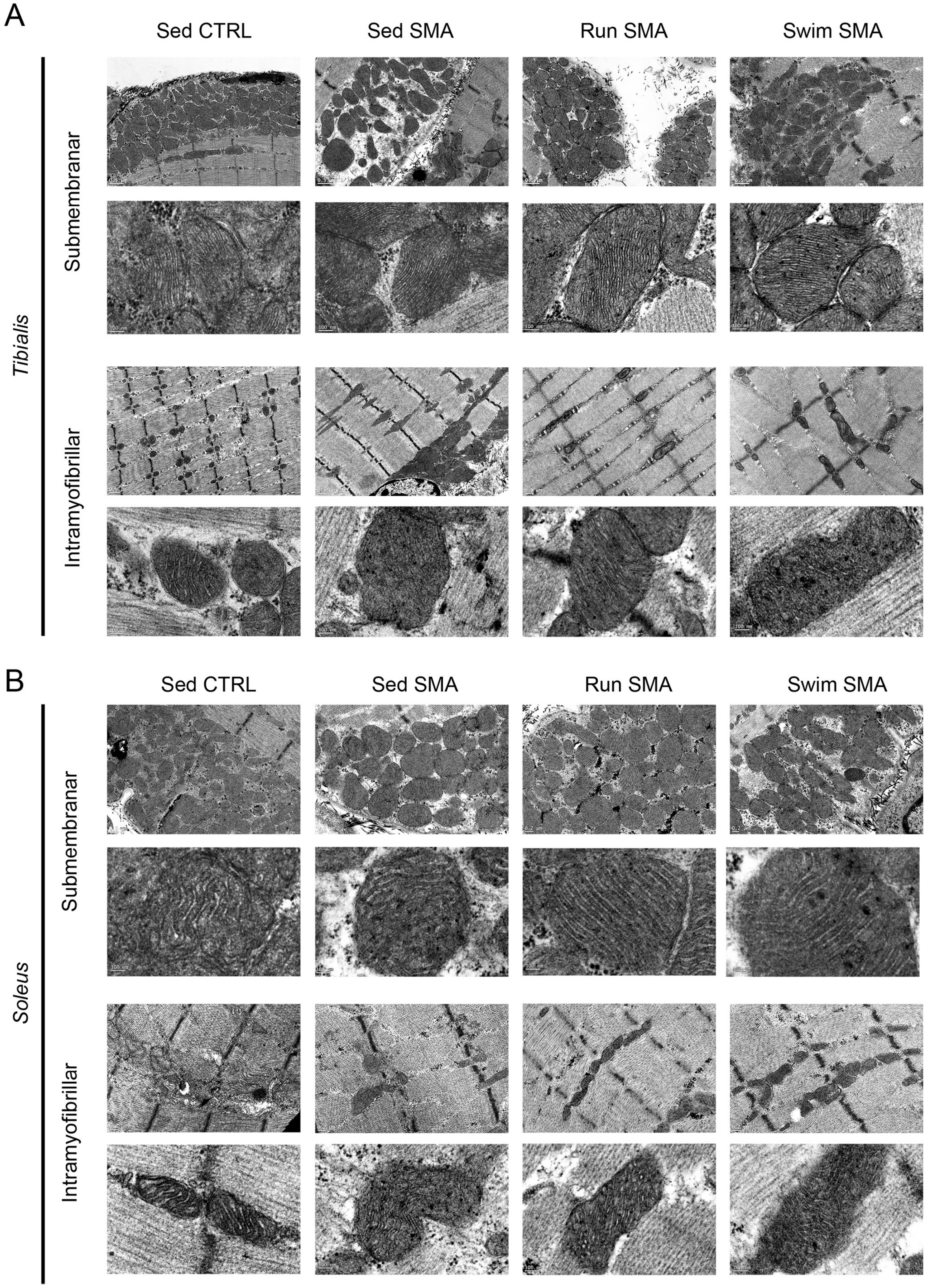

Supplementary Figure S4. (**A, B**) Transmission electron microscopy images of submembranar (up) and intramyofibrillar (down) mitochondria in ultrathin (50-90 nm) *tibialis* (**A**) or *soleus* (**B**) muscles longitudinal sections from 12 months old sedentary control mice compared to sedentary and trained SMA mice (4000X for low magnification and 20 000X for high magnification images).
